# Supplementary material for: How to Measure Foot Self-Care? A Methodological Review of Instruments
Source: J Pers Med. 2023 Feb 28;13(3):434. doi: 10.3390/jpm13030434 (PMC10052648; doi:10.3390/jpm13030434)
Supplement: Supplementary file 1 [file jpm-13-00434-s001.zip › Supplementary Table 1.pdf]

Supplementary Table 1. Descriptive information about the instruments included in the review.

Instrument	Measurement aim	Number of items, subscales/parts	Scoring	Interpretation	Original study		Studies using the instrument	
					Country	Reference	Country	Reference
Diabetes foot self-care behavior scale (DFSBS)	measures frequency of foot self-care behavior	7 items and 2 parts: Part 1) behavior of the participants over one week, 2) frequency of the behaviors exhibited by the participants	5-point Likert scale	A higher score indicates better performance of foot self-care behavior	Taiwan	[30]	Iran<br>China,<br>State of Palestine<br>Malaysia<br>Turkey	[37,45]<br>[36]<br>[38,39]<br>40]
the Summary of Diabetes Self - Care Activities:foot care (SDSCA)	to evaluate self-care behaviour in patients	5 items to identify the number of days in the past week that they performed positive foot care practices (washed their feet, dried between their toes after washing, checked their feet, and inspected the inside of their shoes) or negative foot care practices (soaked feet)	Scaling from 0 to 7 based on the number of days in which the participants indicated their performance	The higher the score, better the foot self-care behaviour	USA	[31]	China<br>Canada,<br>USA<br>Tanzania<br>Brazil<br>South Africa	[46]<br>[47]<br>[48,50]<br>[12]<br>[49,51]<br>[41]
the Nottingham Assessment of Functional Footcare	to assess the foot care behaviour of people with diabetes	29 items: foot care, types of suitable shoes, types of socks, avoiding walking bare foot	Categorical scale (scored 0 to 3) according to the frequency of occurrence of the behaviour	The higher the calculated sum the better foot self-care	UK	[32]	USA<br>Philippines<br>Guyana<br>Egypt	[52]<br>[53]]<br>[54]<br>[43]
Diabetic foot self-care questionnaire of the University of Malaga, Spain (DFSQ-UMA)	to evaluate the foot self-care of diabetic patients	16 questions in 3 domains: Personal self-care (7 items), Podiatry care (4 items) and Shoes and socks (5 items)	4-point response scale		Spain	[33]	Spain	[55]

Foot Self-Care Behaviour (FSCB) questionnaire	To measure foot-care behaviour	17 items in two subscales: Preventive Foot Self-Care (9 items), Potentially Foot – Damaging Behaviour (8 items)	2 different rating scales: a 6 point-scale (i.e. twice a day, daily, every other day, twice a week, once a week or never) and a 4 point-scale (i.e. always, most of the time, occasionally, or never)	A higher score indicates more preventive and potentially damaging behaviours respectively	UK	[34]	Australia	[56–58]
The Foot Self-Care Observation Guide	an objective measurement for the RA who was observing and scoring the foot self-exam components	16 items:<br>Foot care (5 items),<br>Foot check (3 items),<br>Foot safety (8 items)	1 = never, 2 = occasionally, 3 = sometimes, 4 = frequently, and 5 = always.	Range 15–75. Higher scale score indicates that the individual has better self-care behavior	USA	[35]	Turkey	[59]
The Foot Care Knowledge and Practice Questionnaire	N/R	15 questions on knowledge and 15 questions on practice	yes-no		Mexico	[60]		
Modified Diabetic Foot Care Behaviors	Foot self-care behavior				Indonesia	[61]		
Frankfurter Catalogue of Foot Self-Care – Prevention of the Diabetic Foot Syndrome VS. 1.0 (FCFSP)	The FCFSP asks for the health deviation self-care activities for the prevention of diabetic foot syndrome and is based on the Self-Care Deficit Nursing Theory of Orem	19 items in three domains:<br>1: professional assistance in foot care;<br>2: self-control of the feet;<br>3: self-control of shoes and socks.	5-point Likert-scale with a verbal characterisation of the frequency (never, rare, some of the time, most of the time, all the time)		Germany	[62]		
Foot self-care behaviour (FSCB)	foot self-care behavior	16 items in two sections:<br>1: how many days the respondents had performed the foot self-care behaviour in the past seven days (one week, 7 items).	5-point Likert-type scale.	Higher scores representing better foot self-care behaviours. The total score ranged from 16±80	Malaysia	[63]		

		2: the frequency in which respondents performed certain foot self-care behaviour (9 items)						
Foot Self-Care Activities Structured Interview	measures the foot self-care behaviours of older people	16 items in three subscales: 1) general foot self-care (8 items),<br>2) prevention of foot problems (4 items)<br>3) self-care of foot problems (4 items)	multiple-choice response options		Finland	[64]		
Diabetes Foot Disease and Foot Care Questionnaire (DFDFC-Q)	measures foot self-care in diabetic patients	12 items in 6 domains: sociodemographic characteristics, diabetes-related foot disease, foot self-care, footwear, foot care education, and professional foot care	never (0 points), rarely (1 point), once a month (2 points), once a week (3 points), or daily (4 points).	Overall foot selfcare quality score maximum 48 points. Four self-care quality levels are identified: good (37 to 48 points), moderate (25 to 36 points), poor (13 to 24 points), and very poor (12 or fewer points)	Poland	[65]		
Self-foot care Questionnaire	N/R	N/R	N/R		India	[66]		
structured diabetic foot knowledge and practice scoring questionnaire (DiFoKaPS-32)	to measure the subjects' care level of their feet.	16 items:<br>foot self-examination (4 items), footwear (3 items), care of the toenails (2 items), foot hygiene (7 items)	Questions with multiple choice answers were used.		Iran	[67]		
Foot Care History and Assessment of the Feet	N/R	106 item form was developed: 15 demographic and personal health history items; 18 items related to the foot care practices; and 67 assessment items on the condition of the patient's feet	N/R		USA	[68]		

Self-care behavior questionnaire based on the EPPM	N/R	four constructs	N/R		Iran	[69]			
Foot Care Practices Questionnaire	to assess participant's current foot self-care practices	7 items	4 multiple response choises		USA	[70]			
Foot Self-Care Behavior Questionnaire	Performance of foot self-care behaviors	11 items	yes (1) and no (0). The total scale scores ranged from 0 to 11;	higher scores indicated performance of most foot self-care behaviors	Canada	[71]			
the Diabetic Foot Self-care Behavior Scale	evaluating the foot self-care behaviour in diabetic patients.	17 items	4-point Likert scale (1 = never, 2 = sometimes, 3 = often and 4 = always).	Total score range 0–100. A higher score represents a better foot self-care	China	[72]			
"diabetes foot care scale"	N/R	20 items	5 points for each item for a total of 100 points		China	[73]			
Diabetes Care Program of Nova Scotia Diabetes Foot Care Questionnaire	N/R	6 items	yes/no		United Arab emirates	[74]			
Veterans Health Administration Footcare Survey	assessed demographic, general health, diabetes and foot self-care information, barriers to foot self-care, receipt of professional foot care, and satisfaction with current care.	9 items			USA	[75]			
Foot Care Scale for Older Diabetics (FCS-OD)	the ability of older patients with diabetes to self-manage their foot care	9 items (short version) 22 items (long version)	Likert 1-5 (1=very untrue of me - 5=very true of me)		Japan	[76]			

KAP questionnaire	questions about diabetic foot	10 items to evaluate participants' foot care knowledge, 5 items to assess their attitude toward foot care, 8 items to assess their foot care practice	yes/no, in some items multiple choice options		St. Kitts and Nevis	[77]		
Foot Care Confidence/Foot-Care Behavior Scale For Diabetes (FCCS-FCB)	to measure foot care	preventive behaviors (six items) and potentially damaging behaviors (11 items)		Preventive behaviors score range 6–36, high score means the non-observance of preventive behaviors in foot care.<br>Potentially damaging behaviors score range 11– to 44, the higher this score, the more harmful will be the behaviors in foot care.	Iran	[78]		
”no name”	foot self-care was assessed	4 items	yes-no		Brazil	[79]		
“no name”	The questions regarding foot risk factors, self-care behaviors, and education were taken from two instruments, the Diabetes Patient Outcome Research Team Survey and the VA’s Diabetes Quality Improvement Project Survey				USA	[80]		
“no name”	...using selfreports addressing recommended foot self-care among diabetic patients…	4 questions	from "0 days” meant (foot self-care was never performed during the past week), and “7 days”		Iran	[81]		

			meant (foot self-care done every day during the past week)					
”no name”	Knowledge and practice on patients with DM on diabetic foot self-care	16 items (8 for knowledge, 8 for practice)			Ethiopia	[82]		
“no name”	pre-validated questionnaire was used based on the guidelines from The International Working Group on the diabetic Foot and International Diabetes Federation	16 questions, each for knowledge and practice. Questions were asked about daily inspection and washing, proper drying of feet, especially in between the toes, bursting of blisters, care of minor cuts, maintaining water temperature	For most questions yes/no with scores 1 and 0, some items to show the frequency of the task scores ranged from 0 to 4. The cumulative score was 16 for knowledge and 25 for practice.	Interpretation for practice: good practice, 21–25; moderate practice, 14–20; and poor practice, 0–13.	Pakistan	[83]		
“no name” A validated questionnaire in English developed by Hasnain et al. was adopted	to assess the level of foot care knowledge and self-care practices among the respondents	15 items	‘yes’ or ‘no’ questions regarding foot care knowledge and foot self-care practices. Each correct answer was given one mark.	Higher scores signified better foot care knowledge and foot self-care practices.	Malaysia	[84]		
