# Supplementary material for: How to Measure Foot Self-Care? A Methodological Review of Instruments
Source: J Pers Med. 2023 Feb 28;13(3):434. doi: 10.3390/jpm13030434 (PMC10052648; doi:10.3390/jpm13030434)
Supplement: Supplementary file 1 [file jpm-13-00434-s001.zip › Supplementary Table 2.pdf]

Supplementary Table 2. Methodological quality of included studies (n=53) using MMAT tool.

	Reference	**RCT-quantitative**							**Non-random quantitative**							**Quantitative**						
		**s1**	**s2**	**2_1**	**2_2**	**2_3**	**2_4**	**2_5**	**s1**	**s2**	**3_1**	**3_2**	**3_3**	**3_4**	**3_5**	**s1**	**s2**	**4_1**	**4_2**	**4_3**	**4_4**	**4_5**
DFSBS	Chin & Huang, 2013 [30]															Y	Y	Y	Y	Y	Y	Y
	Bahador et al., 2017 [37]								Y	Y	Y	Y	Y	N	Y							
	Chin et al., 2014 [45]															Y	Y	Y	Y	Y	Y	Y
	Salameh et al., 2020 [36]															Y	Y	Y	Y	Y	N	Y
	Ahmad Sharoni et al., 2017b [39]															Y	Y	Y	Y	Y	Y	Y
	Ahmad Sharoni et al., 2017a [38]								Y	Y	Y	Y	Y	Y	Y							
	Sahin & Cingil, 2020 [40]															Y	Y	Y	Y	Y	N	Y
SDSCA	Toobert et al., 2000 [31]															Y	Y	Y	Y	Y	Y	Y
	Li et al., 2014 [46]															Y	Y	Y	Y	Y	Y	Y
	Al Sayah et al., 2015 [47]															Y	Y	Y	Y	Y	Y	Y
	Bell et al., 2005 [48]															Y	Y	Y	Y	Y	N	Y
	Chiwanga, 2015 [12															Y	Y	Y	Y	Y	N	Y
	Rezende Neta et al., 2015 [49]															Y	Y	Y	Y	Y	N	Y
	Jordan & Jordan, 2011 [50]															Y	Y	Y	Y	Y	N	Y
	Dikeukwu & Omole, 2013 [41]															Y	Y	Y	Y	Y	Y	Y
	Batista et al., 2020 [51]															Y	Y	Y	Y	Y	Y	Y
NAFF	Lincoln et al., 2007 [32]															Y	Y	Y	Y	Y	Y	Y
	Wendling et al., 2015 [52]															Y	Y	Y	Y	Y	Y	Y
	Magbanua & Lim-Alba 2017 [53]															Y	Y	Y	Y	Y	N	Y
	Kurup et al., 2019 [54]															Y	Y	Y	Y	Y	N	Y
	Abdelhamid et al., 2018 [43]	Y	Y	Y	Y	Y	N	Y														
DFSQ-UMA	Navarro-Flores et al., 2015 [33]															Y	Y	Y	Y	Y	Y	Y
	Gonzalez-de la Torre et al., 2020 [55]															Y	Y	Y	Y	Y	N	Y
FSCB	Vileikyte et al., 2006 [34]															Y	Y	Y	Y	Y	N	Y
	Nguyen et al., 2019 [56]								Y	Y	Y	Y	Y	Y	Y							
	Perrin et al., 2009 [57]															Y	Y	Y	Y	Y	N	Y
	Perrin et al., 2014 [58]															Y	Y	Y	Y	Y	N	Y
FSOG	Borges & Ostwald, 2008 [35]	Y	Y	Y	Y	Y	Y	Y														
	Gökdeniz & Akgün Şahin, 2020 [59]															Y	Y	Y	Y	Y	N	Y
Other	Bohorquez Robles et al., 2017 [60]															Y	Y	Y	Y	Y	N	Y

	Sari et al., 2020 [61]															Y	Y	Y	Y	Y	N	Y
	Schmidt et al., 2008 [62]															Y	Y	Y	Y	Y	N	Y
	Ahmad Sharoni et al., 2018 [63]	Y	Y	Y	Y	Y	Y	Y														
	Stolt et al., 2012 [64]															Y	Y	Y	N	N	Y	Y
	Świątoniowska et al., 2020 [65]															Y	Y	Y	Y	Y	N	Y
	Thomas et al., 2017 [66]															Y	Y	Y	Y	Y	N	Y
	Vatankhah et al., 2009 [67]								Y	Y	Y	Y	Y	N	Y							
	Kruger & Guthrie, 1992 [68]															Y	Y	Y	Y	Y	Y	Y
	Lael-Monfared et al., 2019 [69]															Y	Y	Y	Y	Y	N	Y
	Corbett, 2003 [70]	Y	Y	Y	Y	Y	Y	Y														
	Fan et al., 2014 [71]								Y	Y	Y	Y	Y	N	Y							
	Li et al., 2019 [72]								Y	Y	Y	Y	Y	N	Y							
	Liang et al., 2012 [73]															Y	Y	N	N	Y	Y	Y
	Makiling & Smart, 2020 [74]								Y	Y	Y	Y	Y	N	Y							
	Olson et al., 2009 [75]															Y	Y	Y	Y	Y	Y	Y
	Sable-Morita et al., 2021 [76]															Y	Y	Y	Y	N	N	Y
	Hanley et al., 2020 [77]															Y	Y	Y	Y	Y	Y	Y
	Ghasemi et al., 2021 [78]	Y	Y	Y	Y	Y	N	Y								Y	Y	Y	Y	N	N	Y
	Rossaneis et al., 2016 [79]															Y	Y	Y	Y	Y	N	Y
	Madarshanian et al. 2014 [81]															Y	Y	Y	Y	N	N	Y
	Tuha et al., 2021 [82]															Y	Y	Y	Y	N	N	Y
	Qasim et al., 2021 [83]															Y	Y	Y	Y	Y	N	Y
	Ong et al., 2022 [84]															Y	Y	Y	Y	N	N	Y

Y = yes, N = no; DFSBS = Diabetes foot self-care behavior scale, SDSCA = The Summary of Diabetes Self - Care Activities: foot care; NAFF = the Nottingham Assessment of Functional Footcare; DFSQ-UMA = diabetic foot self-care questionnaire of the University of Malaga, Spain; FSCB = Foot Self-Care Behaviour questionnaire; FSOG = The Foot Self-Care Observation Guide
