# Supplementary material for: How to Measure Foot Self-Care? A Methodological Review of Instruments
Source: J Pers Med. 2023 Feb 28;13(3):434. doi: 10.3390/jpm13030434 (PMC10052648; doi:10.3390/jpm13030434)
Supplement: Supplementary file 1 [file jpm-13-00434-s001.zip › Supplementary Table 3.pdf]

Supplementary Table 3. Detailed analysis of psychometric evidence of six instruments measuring foot self-care, analysed against the criteria proposed by Zwakhalen and colleagues [28].

Instrument	Origin of items (0-2)		Num. of particip. (0-2)		Validity										Reliability						Total score (0–20)
					Content (0-2)		Criterion (0-2)		Construct I: in relation to other tests (0-2)		Construct II: differentiates (0-2)		Homogeneity (0-2)		Intra-rater (0-2)		test-retest (0-2)		Feasibility (0-2)		
**Diabetes foot self-care behavior scale (DFSBS)**																					
Diabetes foot self-care behavior scale (DFSBS) [30]	developed and tested in this study, a comprehensive review of the literature to identify diabetic patient foot care guidelines and existing foot self-care behavior scales.	**2**	295 patients with diabetes and peripheral neuropathy	**2**	experts rated the adequacy and clarity, content validity index	**2**	correlation with Chinese version of the Summary of Diabetes Self-care Activity questionnaire	**2**	Exploratory factor analysis, convergent validity analysis, known groups validity analysis	**2**	item analysis, item discrimination index	**2**	Cronbach's alpha 0.73.	**2**	N/R	**0**	N/R	**0**	A pilot study	**2**	**16**
Use of DFSBS in Iran [37]	N/R	**2**	60 patients with diabetic foot ulcers	**2**	content validity with ten faculty members	**2**	N/R	**0**	N/R	**0**	N/R	**0**	Cronbach's alpha 0.86.	**2**	N/R	**0**	N/R	**0**	N/R	**0**	**8**
Use of DFSCBS in Taiwan [45]	N/R	**2**	290 patients with diabetes	**2**	N/R	**0**	N/R	**0**	N/R	**0**	N/R	**0**	Cronbach's alpha 0.73	**2**	N/R	**0**	N/R	**0**	N/R	**0**	**6**
Use of DFSBS in State of Palestine [36]	DFSBS was translated into Arabic	**2**	413 patients with diabetes	**2**	content evaluated by three experts in the field	**2**	N/R	**0**	N/R	**0**	N/R	**0**	Cronbach's Alpha 0.83	**2**	N/R		N/R	**0**	A pilot study with 10 patients with diabetes	**2**	**10**

Use of DFSBS in Malaysia [39]	Refer to original study [30]	**2**	126 older people with diabetes	**2**	panel of four experts	**2**	N/R	**0**	N/R	**0**	N/R	**0**	N/R	**0**	N/R	**0**	N/R	**0**	N/R	**0**	**6**
Use of DFSBS in Turkey [40]	Refer to original study [30]	**2**	246 patients with diabetes	**2**	N/R	**0**	N/R	**0**	N/R	**0**	N/R	**0**	Cronbach’s alpha 0.50.	**1**	N/R	**0**	N/R	**0**	N/R	**0**	**5**
Use of DFSBS in Malaysia [38]	Refer to original study [30] and official forward and backward translations from English to Malay and back	**2**	31 older people with diabetes	**2**	Content validity ratio (CVR) assessed by six experts	**2**	N/R	**0**	N/R	**0**	N/R	**0**	Cronbach's alpha 0.68.	**1**	N/R	**0**	N/R	**0**	pilot study	**2**	**9**
**the Summary of Diabetes Self - Care Activities (SDSCA): foot care**																					
Use of SDSCA in United States [48]	Refer to original study [31]	**2**	688 older adults with type 2 diabetes	**2**	N/R	**0**	N/R	**0**	N/R	**0**	N/R	**0**	N/R	**0**	N/R	**0**	N/R	**0**	N/R	**0**	**4**
Use of SDSCA in Tanzania [12]	Refer to original study [31]	**2**	404 patients with diabetes	**2**	N/R	**0**	N/R	**0**	N/R	**0**	N/R	**0**	N/R	**0**	N/R	**0**	N/R	**0**	N/R	**0**	**4**
Use of SDSCA in Brazil [49]	translated and adapted version for the Brazilian culture	**0**	331 patients with diabetes	**2**	N/R	**0**	N/R	**0**	N/R	**0**	N/R	**0**	N/R	**0**	N/R	**0**	N/R	**0**	N/R	**0**	**2**
Use of SDSCA in Filippines [50]	Refer to original study [31]	**2**	118 patients with diabetes	**2**	N/R	**0**	N/R	**0**	N/R	**0**	N/R	**0**	N/R	**0**	N/R	**0**	N/R	**0**	N/R	**0**	**4**
Use of SDSCA in South Africa [41]	Refer to original study [31]	**2**	120 patients with diabetes	**2**	N/R	**0**	N/R	**0**	N/R	**0**	N/R	**0**	N/R	**0**	N/R	**0**	N/R	**0**	pilot	**2**	**6**
the Diabetes Self-Care Activities Questionnaire (DSQ) [51]	version translated and adapted to the Brazilian culture	**2**	197 patients with diabetes	**2**	N/R	**0**	N/R	**0**	N/R	**0**	N/R	**0**	N/R	**0**	N/R	**0**	N/R	**0**	N/R	**0**	**4**
**Nottingham Assessment of Functional Foot-care questionnaire (NAFF)**																					

the Nottingham Assessment of Functional Footcare, original study [32]	previous literature and foot care recommendations and guidelines	**2**	128 patients with and without diabetes	**2**	N/R	**0**	N/R	**0**	N/R	**0**	convergent validity	**2**	0.53	**1**	N/R	**0**	test-retest	**2**	pilot	**2**	**11**
The use of NAFF in United Kingdom [42]	Refer to original study [32]	**2**	90 patients with diabetes	**2**	N/R	**0**	N/R	**0**	N/R	**0**	convergent validity	**2**	0.61	**1**	N/R	**0**	test-retest	**2**	N/R	**0**	**9**
The use of NAFF in United Kingdom [52]	Refer to original study [32]	**2**	223 patients with diabetes	**2**	N/R	**0**	N/R	**0**	N/R	**0**	N/R	**0**	N/R	**0**	N/R	**0**	N/R	**0**	N/R	**0**	**4**
The use of NAFF in United Kingdom [43]	a modified version of the Nottingham Assessment of Functional Foot-care questionnaire (NAFF)	**2**	control (n = 35), interventio n (n = 35), patients with diabetes	**2**	panel of three experts for assesse d face and content validity	**2**	N/R	**0**	N/R	**0**	N/R	**0**	Cronbach’s alpha 0.891 .	**2**	N/R	**0**	N/R	**0**	N/R	**0**	**8**
**Diabetic foot self-care questionnaire of the University of Malaga, Spain (DFSQ-UMA)**																					
diabetic foot self-care questionnaire of the University of Malaga, Spain (DFSQ-UMA) [33]	developed in this study based on literature	**2**	209 patients with diabetes	**2**	expert panel	**2**	The correlation with HbA1c was significant	**2**	Exploratory factor analysis	**2**	Inter-item and item-to-total correlation s.	**2**	Cronbach’s alpha 0.89.	**2**	N/R	**0**	test-retest reliabilit y	**2**	congitive interview s of item clarity	**2**	**18**
The use of DFSQ-UMA in Spain [55]	Refer to original study [33]	**2**	182 patients with diabetes	**2**	N/R	**0**	N/R	**0**	N/R	**0**	N/R	**0**	N/R	**0**	N/R	**0**	N/R	**0**	N/R	**0**	**4**
**Foot Self-Care Behaviour (FSCB) questionnaire**																					
The use of FSCB in Australia [56]	Refer to original study [34]	**2**	119 people with type 2 diabetes at low risk of developin	**2**	excellen t content validity index (CVI)	**2**	N/R	**0**	N/R	**0**	N/R	**0**	Cronbach alpha 0.96 and 0.68	**2**	N/R	**0**	N/R	**0**	N/R	**0**	**8**

			g a foot ulcer																		
The use of FSCB in Australia [57]	a self-report questionnaire derived from a tool developed by Vileikyte and colleagues	**2**	96 people with diabetes	**2**	N/R	**0**	N/R	**0**	N/R	**0**	N/R	**0**	N/R	**0**	N/R	**0**	N/R	**0**	N/R	**0**	**4**
**The Foot Self-Care Observation Guide**																					
Original study [35]	developed for this study to reflect the content of American Diabetes Association guidelines	**2**	144	**2**	A podiatrist reviewed the instrument	**1**	N/R	**0**	N/R	**0**	N/R	**0**	Cronbach's alpha 0.68	**1**	N/R	**0**	test-retest	**2**	pilot test	**2**	**10**
Gökdeniz & Sahin 2020 [59]	Refer to original study [35]	**2**	120	**2**	N/R	**0**	N/R	**0**	N/R	**0**	N/R	**0**	Cronbach's alpha 0.83	**2**	N/R	**0**	N/R	**0**	N/R	**0**	**6**
